# *Gossypium hirsutum* gene of unknown function Gohir.A02G131900 encodes a potential plant-specific, dual-domain exo-1,3-β-glucosidase

**DOI:** 10.17912/micropub.biology.000868

**Published:** 2024-01-28

**Authors:** Gillian Hernandez, Amanda M Hulse-Kemp, Amanda R Storm

**Affiliations:** 1 Department of Biology, Western Carolina University, Cullowhee, NC USA; 2 Genomics and Bioinformatics Research Unit, USDA-ARS, Raleigh, NC; 3 Department of Crop and Soil Sciences, North Carolina State University, Raleigh, NC

## Abstract

A gene of unknown function, Gohir.A02G131900.1, identified in *Gossypium hirsutum* was studied using computational sequence and structure bioinformatic tools. The encoded protein GhGH5BG-A0A1U8NW40 (UniProt A0A1U8NW40) is predicted to be secreted and localized to the cell wall. Homology and conserved residues indicate it belongs to a plant-specific subgroup of the glycoside hydrolase family 5 and likely has exo-1,3-β-glucosidase activity. This subgroup is unique in containing a fascin-like domain which may have evolved a unique glucan binding site of interest for further research.

**Figure 1. Sequence and Structure Characterization of GhGH5BG-A0A1U8NW40:**
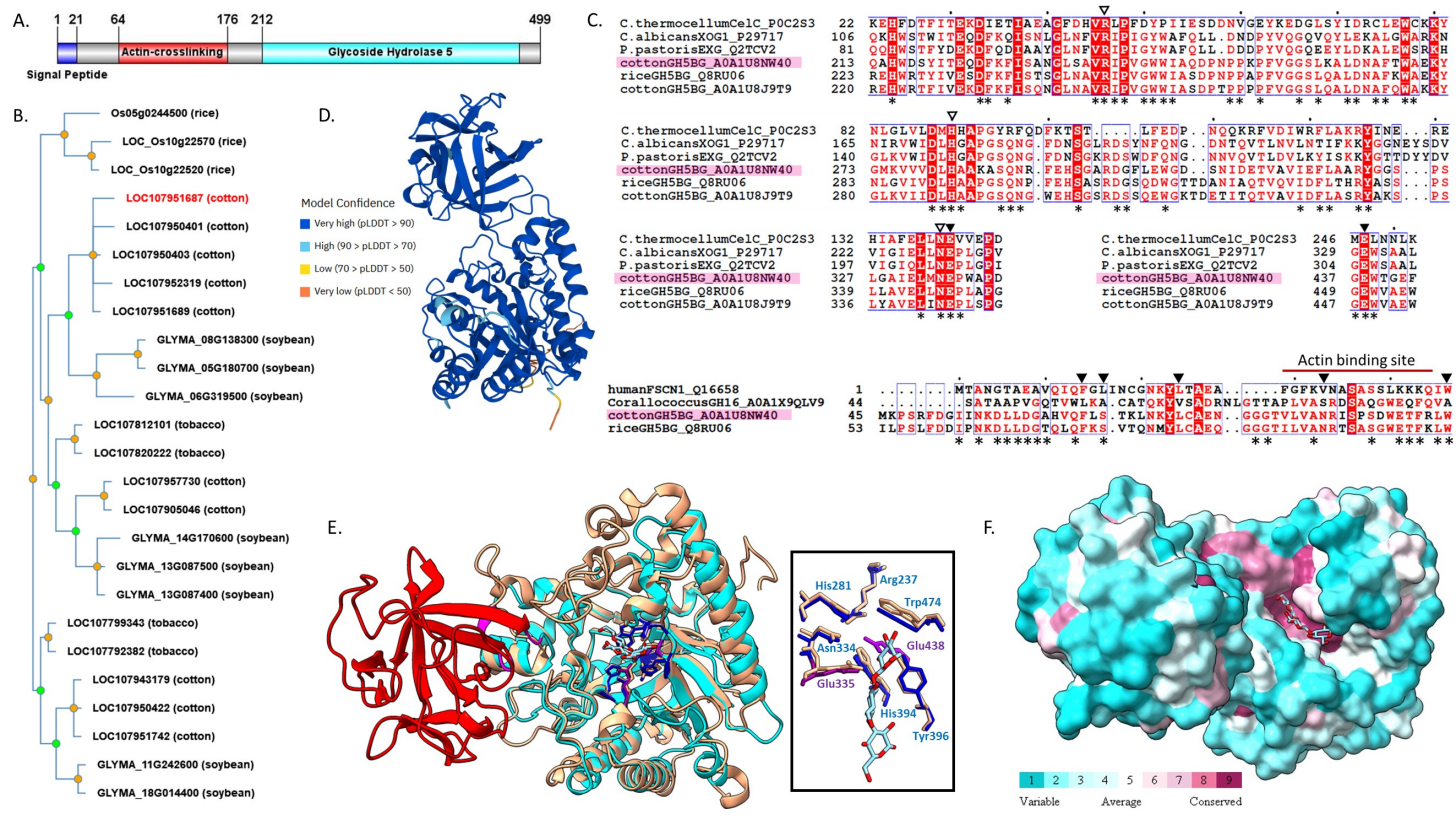
( **A** ) Domain architecture of *Gossypium hirsutum* Glycosyl Hydrolase 5 β-Glucosidase (GhGH5BG-A0A1U8NW40) depicting the predicted signal peptide, actin-crosslinking (fascin-like) domain and glycoside hydrolase 5 (GH5) domain, created with Illustrator for Biological Science (Liu et al. 2015) using predictions from InterPro and BUSCA. ( **B** ) Phylogenetic tree of *Gossypium hirsutum* GhGH5BG-A0A1U8NW40 and homologs from soybean, tobacco, rice, and corn, created by PhyloGenes, GhGH5BG-A0A1U8NW40 sequence highlighted in red. ( **C** ) Top: Multi-sequence alignment of catalytic regions of glycoside hydrolase 5 domain containing sequences of GhGH5BG-A0A1U8NW40 (pink shading), exo-1,3-β-glucosidases XOG1 from *Candida albicans* (P29717) and EXG from *Pichia pastoris* (Q2TCV2), endo-1,4-β-glucosidase from *Clostridium thermocellum* (P0C2S3), GH5BG from rice (Q8RU06), and cotton paralog (A0A1U8J9T9). Invariant residues of GH5 family indicated by empty triangles, catalytic glutamate residues indicated by filled triangles (Opassiri et al. 2007), asterisks (*) indicate ConSurf-predicted highly conserved residues in GhGH5BG-A0A1U8NW40. Bottom: MSA of fascin-like (actin-crosslinking) domain of GhGH5BG-A0A1U8NW40 aligned with N-terminal subdomain 1 of human fascin-1 (Q16658) and the fascin-like domain of rice GH5BG (Q8RU06) and *Corallococcus* sp. strain EGB GH16 LamC (A0A1X9QLV9). Annotations indicate actin binding site (red bar) and β-trefoil stabilizing residues (triangles) identified in human fascin-1 (Sedeh et al. 2010) as well as ConSurf-predicted highly conserved residues in GhGH5BG-A0A1U8NW40 (asterisks). Alignments created using ClustalOmega and ESPript3 ( **D** ) AlphaFold model structure of GhGH5BG-A0A1U8NW40, depicting model confidence. ( **E** ) Structure overlay of GhGH5BG-A0A1U8NW40 (GH5 domain: cyan; actin-crosslinking domain: red) and homologous exo-1,3- β-glucosidase XOG1 (PDB ID 3N9K) from *C. albicans* (cream ribbon), created using ChimeraX MatchMaker. Stick representation of substrate analog laminaritriose (element coloring) and selected side chains (GH5-invariant residues: blue ; catalytic residues: purple; Opassiri et al. 2007); backbones hidden in inlay. (F) GhGH5BG-A0A1U8NW40 with overlaid laminaritriose showing ConSurf conservation coloring.

## Description

Introduction

Recently, the genomes of five cotton species were analyzed with the objective of understanding genes that are involved in agriculturally relevant traits (Chen et al. 2020) . Protein functions were not able to be automatically predicted for thousands of genes found in these species. In the sequencing of the genome of upland cotton, *Gossypium hirsutum* , the gene LOC107951687 (Gohir.A02G131900.1_UTX-TM1_v2.1, CottonGen: https://www.cottongen.org/bio_data/5890412 ) codes for a protein of unknown function identified as ‘probable glucan 1,3-beta-glucosidase A isoform X1’ (UniProt A0A1U8NW40; NCBI XP_016742298). Here we present computational sequence and structure analysis of this protein, referred to here as GhGH5BG-A0A1U8NW40 ( *Gossypium hirsutum* Glycosyl Hydrolase 5 β-Glucosidase), that supports this protein being labeled an exo-1,3-β-glucosidase within the glycosyl hydrolase 5 (GH5) family and belonging to a plant-specific subgroup containing a unique fascin-like (actin-crosslinking) domain.

The GH5 family contains enzymes with several known activities including endo-glucosidases (also known as glucanases), beta-mannanases, exo-1,3-glucosidases and xylanases (Opassiri et al. 2007) . Glucan 1,3- β-glucosidases are exo-glucanases that catalyze hydrolysis of beta-D-glucose units from the non-reducing ends of 1,3-β-D-glucans (EC 3.2.1.58) and members have been shown to be part of the oligosaccharide degradation in plant cell walls (Singh et al. 2015) . Proteins with a GH5 domain have a protein structure with an alpha-beta (β/α) _8_ or TIM (triose-phosphate isomerase) barrel, commonly found in protein catalysts (Silverman et al. 2001) .

In addition to the GH5 domain, the GhGH5BG-A0A1U8NW40 protein has a second domain with structural homology to a Fascin subdomain. Fascin domains are found in a family of actin-binding proteins across invertebrate and vertebrate eukaryotes. Fascin is involved in the organization of the actin cytoskeleton and cell motility, crosslinking actin within filamentous actin filopodia structures (Yamashiro et al. 1998) . The structure of fascin family proteins consists of four tandem β-trefoil fold subdomains (Sedeh et al. 2010) .

No homologs of fascin proteins are found in plants but proteins containing a glucosidase domain and a single β-trefoil fold fascin-like subdomain have been identified in plants and bacteria (Tyler et al. 2010, Zhou et al. 2017) . One of these proteins with exo-1,3-β-glucosidase and fascin-like domains was studied in rice (Opassiri et al. 2007) . The expression of this protein was highest in seedling shoots and mature leaves with induction under certain stresses. The protein was found to be secreted, which is a possible indication of it being involved in cell wall remodeling. The rice glycosyl hydrolase 5 β-glucosidase (GH5BG) has substrate specificity with a preference for 1,4-β-linked oligosaccharides and laminaribiose, differing from fungal GH5 exo-1,3- β-glucosidase. It was proposed that this rice GH5BG protein was part of a plant-specific subgroup of the GH5 exo-glucosidase subfamily (Opassiri et al. 2007) . This is supported by a comparative study of the glycoside hydrolase 5 genes in plants which were found to be divided into 3 major clades (A, B, and C). All proteins belonging to Clade B, including the rice GH5BG, contain a predicted fascin-like subdomain (Tyler et al. 2010) . Pairing of a fascin-like subdomain with a glycosyl hydrolase domain was also reported in some bacterial proteins, including a GH16 1,3-β-glucanase LamC in *Corallococcus* sp. EGB, which was studied using *in vitro* activity assays. In this *Corallococcus* protein, the fascin-like domain was found to not bind actin; however, removal of this domain from LamC reduced binding and activity towards 1,3-β-linked glucans (Zhou et al. 2017) .

Sequence features

InterPro database (Blum et al. 2020) identified sequence features for the 499 amino acid GhGH5BG-A0A1U8NW40 protein that associate the protein as part of both the ‘actin-crosslinking’ (IPR008999) and ‘glycoside hydrolase’ (IPR017853) superfamilies based on features in N-terminal and C-terminal regions, respectively. It was placed in the ‘Fascin’ family (IPR010431) with an identified ‘glycoside hydrolase (Cellulase A) family 5’ domain (IPR001547). The subcellular targeting peptide program Plant-mPloc (Chou and Shen 2010) predicted GhGH5BG-A0A1U8NW40 localizes to the cell wall and the BUSCA (Savojardo et al. 2018) program predicted a secretion signal peptide at amino acids 1-21. A domain architecture shows the location of these features of GhGH5BG-A0A1U8NW40 ( **Figure 1A** ).

Homology

The PhyloGenes program (Zhang et al. 2020) produced a phylogenetic tree for GhGH5BG-A0A1U8NW40 with 170 homologs from 38 plant and non-plant organisms. Non-plant homologs were homologous across only the N-terminal fascin-like (actin-crosslinking) domain. No homologous proteins were found in *Arabidopsis thaliana* either through PhyloGenes or direct searching of the TAIR database with BLASTp. Homologs of GhGH5BG-A0A1U8NW40 were found in other agricultural plant species including soybean, tobacco, rice, and maize. The PhyloGenes phylogenetic tree of these homologs revealed three clusters containing cotton homologs with GhGH5BG-A0A1U8NW40 clustering closest with a set of soybean homologs ( **Figure 1B** ). Data from UniProt showed each of these proteins had the same domain, family, GO terms, and extracellular predicted subcellular location, but lacked a known function. The previously studied rice GH5BG homolog (Os10g22520, Opassiri et al. 2007) was not found in the same cluster as GhGH5BG-A0A1U8NW40.

As the GH5 family contains a diversity of enzymatic activities, a multi-sequence alignment was created to compare GhGH5BG-A0A1U8NW40 from *G. hirsutum* with exo-1,3-β-glucosidases from *C. albicans* (XOG1) and *P. pastoris* (Exg), an endo-1,4-β-glucosidase from *C. thermocellum* (celC), the GH5BG from rice (Q8RU06), and a cotton paralog (A0A1U8J9T9) from another cluster. A portion of the alignment is shown for the regions surrounding the catalytic residues in the GH5 domain ( **Figure 1C** , top, full MSA in Extended Data). A previous study comparing the rice GH5BG with other exo-1,3-β-glucosidases (Opassiri et al. 2007) identified six invariant residues characterizing the GH5 family, some of which are shown in the alignment region ( **Figure 1C** , empty triangles) in addition to the two catalytic glutamates ( **Figure 1C** , filled triangles). The invariant residues formed hydrogen-bond interactions to the non-reducing terminal sugar in the -1 subsite of *C. albicans* XOG1 (Cutfield et al. 1999) *.* All eight residues are conserved in GhGH5BG-A0A1U8NW40 including the motifs surrounding the catalytic acid/base glutamate “NEP” (Glu335 in GhGH5BG-A0A1U8NW40) and catalytic nucleophile glutamate “GEW” (Glu438 in GhGH5BG-A0A1U8NW40). The close similarity of GhGH5BG-A0A1U8NW40 to the rice GH5BG (Q8RU06) (70% similarity by BLASTp) indicates this cotton homolog belongs to the same proposed plant-specific GH5 subgroup.

The sequence of the fascin-like (actin-crosslinking) domain of GhGH5BG-A0A1U8NW40 was aligned with the N-terminal β-trefoil subdomain 1 of human Fascin-1 and the fascin-like domain of the rice GH5BG and *Corallococcus* GH16 LamC ( **Figure 1C** , bottom). Some residues that stabilize the core of the β-trefoil fold (triangles, Sedeh et al. 2010) were shared among most sequences. However, the identified actin binding site in subdomain 1 of human fascin-1 (red bar) is largely not conserved in the bacterial or plant sequences suggesting that this region has developed a new function in proteins with the fascin-like subdomain. This is in agreement with experimental findings from the bacterial LamC study (Zhou et al. 2017) where no actin binding was found and the secreted, cell wall location of the plant homologs also suggests a different function as actin is primarily located in the cytosol (Opassiri et al. 2007) .

Structural features

The predicted structure of GhGH5BG-A0A1U8NW40 created by AlphaFold (Jumper et al. 2021) was used to visualize and analyze the molecular structure ( **Figure 1D** ). The highest region of confidence within the structure was between amino acids 26-499 and the lowest confidence region was at the N-terminus, including the predicted signal peptide region from amino acid 1-25, which was deleted from the structure for subsequent analyses. The overall structure of GhGH5BG-A0A1U8NW40 showed two folded domains, where one domain consists of the GH5 domain (residues 25-53 and 194-495), and the other is the fascin-like subdomain (residues 64-176). The GH5 domain of the GhGH5BG-A0A1U8NW40 protein shows the typical structure of a (β/α) _8_ barrel, which is a structure of eight alpha helices and eight parallel beta strands. The structure of the fascin-like subdomain consists of 10 beta sheets and 1 partial alpha helix in the arrangement of a β-trefoil fold found in fascin proteins (Sedeh et al. 2010) .

Although binding sites in GhGH5BG-A0A1U8NW40 are not known, its structure can be compared to other studied homologs. The RCSB PDB database (Berman et al. 2000) was searched for proteins homologous to GhGH5BG-A0A1U8NW40 and results contained GH5 protein mostly from pathogenic yeast *Candida albicans* . The closest homolog with a known structure is a *C. albicans* exo-1,3-β-glucosidase (PDB 3N9K) with a 29% sequence identity. This homolog has been studied experimentally and structurally (including PDB 1CZ1, 1EQP, 2PB1, 4M8O) and was determined to be located within the cell wall and involved in cell wall glucan metabolism, catalyzing the hydrolytic removal of a glucose residue from the non-reducing end of the 1,3-β-glucan (Cutfield et al. 2000; Patrick et al. 2010) . A structure overlay was created with 3N9K and the GhGH5BG-A0A1U8NW40 model structure ( **Figure 1E** ) showing the structural similarity over the GH5 domain (rmsd of 0.943 angstroms across 211 pruned atom pairs). The 3N9K structure contains a substrate analog, laminaritriose, that models into a deep pocket within the GH5 domain in the overlay. Residues reported to be invariant in GH5 enzymes and the two catalytic glutamate residues are all conserved in GhGH5BG-A0A1U8NW40 and the placement of these residues in the model align with the 3N9K structure ( **Figure 1E** , inlay) beyond one catalytic Glu that is missing from the 3N9K structure due to mutagenesis. Such information supports the prediction that GhGH5BG-A0A1U8NW40 is a catalytically active GH5 enzyme. ConSurf was used to obtain the conservation of residues in GhGH5BG-A0A1U8NW40 and the residues in the proposed pocket binding site were all highly conserved ( **Figure 1F** ). None of the structures identified by searching PDB or DALI databases with GhGH5BG-A0A1U8NW40 contained a fascin-like subdomain.

The GH5 family contains both exo and endo glucosidases, which are very similar in structure and mechanism. The proposed function of GhGH5BG-A0A1U8NW40 as an exo (1,3-β-glucosidase) rather than an endo (cellulase) enzyme is supported by the structure model containing a deep pocket characteristic of enzymes that degrade polysaccharides from the end, rather than an open groove as seen in endo enzymes (Cutfield et al. 1999) . This is also supported by searching DALI (Holm 2020) for structure matches to the GhGH5BG-A0A1U8NW40 model where the closest structural analogs were exo-1,3-β-glucosidases (PDB 3N9K, Z score 42.0, rmsd 2.0) rather than cellulases (PDB 1CEN, Z score 25.8, rmsd 2.7). Additionally, structural analysis of *C. albicans* exo-1,3-β-glucosidase indicated the importance of Glu27 in substrate binding (Cutfield et al. 1999) . This Glu is conserved across GH5 exo-glucosidases, but not endo-glucosidases, and GhGH5BG-A0A1U8NW40 has a highly conserved Glu at the corresponding position (ConSurf results in Extended Data).

Conclusion

The structural and sequence data observed from homolog structures and conserved regions provide evidence that GhGH5BG-A0A1U8NW40 is a member of a plant-specific subgroup of the glycoside hydrolase family 5 with exo-1,3-β-glucosidase activity. The subcellular localization results predict the secretion of GhGH5BG-A0A1U8NW40 to the cell wall location. The conserved GH5 family residues in GhGH5BG-A0A1U8NW40 and a deep pocket binding site similar to active exo-1,3-β-glucosidases indicate that there is a catalytic site with all the residues required to hydrolyze glucans in the cell wall metabolic process (Minic & Jouanin 2006) . The additional fascin-like subdomain within GhGH5BG-A0A1U8NW40 still elicits unanswered questions as to its possible function, although evidence suggests it may have evolved a unique glucan binding site worth further research.

## Extended Data

Description: ConSurf sequence conservation results. Resource Type: Dataset. DOI: 10.22002/rm45z-4k762

Description: Full MSA for Figure 1c GH 5 alignment. Resource Type: Dataset. DOI: 10.22002/14cdc-x8v09

Description: Full MSA for Figure 1c Fascin alignment . Resource Type: Dataset. DOI: 10.22002/vqckk-6vv91
